# Framework for assessing vertebrate invasive species damage: the case of feral swine in the United States

**DOI:** 10.1007/s10530-020-02311-8

**Published:** 2020-07-10

**Authors:** Stephanie Shwiff, Alex Pelham, Steven Shwiff, William Haden-Chomphosy, Vienna R. Brown, Karina Ernst, Aaron Anderson

**Affiliations:** 1 USDA/APHIS/WS National Wildlife Research Center, Fort Collins, CO USA; 2 University of Massachusetts Boston, Boston, MA USA; 3 Texas A&M, Commerce, TX USA; 4 Hendrix College, Conway, AR USA; 5 USDA/APHIS/WS National Feral Swine Damage Management Program, Fort Collins, CO USA

**Keywords:** Depredation, Destruction, Disease, Economics, Feral swine, Vertebrate invasive species

## Abstract

The aim of this study is to provide a general overview of the economic impacts associated with vertebrate invasive species (VIS) in the United States and suggests a methodology for differentiating types of damage. We identify a general framework for categorizing VIS damage that separates this damage into three main categories: destruction, depredation, and disease. We then examine how this framework fits into current published estimates of damage and management costs. Economic impacts associated with feral swine damage and management are plentiful enough to warrant separate treatment from other VIS and are observed in all three categories. For all VIS examined in this study, damage estimates associated with destruction provide the most evaluations of VIS impacts, especially destruction of crops. Evaluations of the losses associated with depredation are largely absent from the literature. We find that while published studies have estimated substantial economic impact associated with VIS, the current state of the literature focusing on VIS frequently fails to address all of the categories of damage, is difficult to compare or replicate, and is unsuited for extrapolation to nation-wide estimates of damage.

## Introduction

Invasive species are a persistent and significant source of economic loss within the United States. With annual damage estimates exceeding $100 billion, these species have become a leading cause of environmental change and global biodiversity loss (Wilcove et al. 1998; Mack et al. 2000; Sala et al. 2000; Pimentel et al. 2005). Harmful non-native species highlight the undeniable link and feedback loops between ecology and economics (Perrings et al. 2002; Julia et al. 2007). Economic systems, for example the exotic pet trade, are a primary route of introduction for non-native species, and ecological systems determine whether or not an environment is susceptible to invasion by one of those species. Invasive species diminish the ability of ecosystems to provide services, such as water filtration and forage coverage, and often render livestock and crops to be unmarketable (Julia et al. 2007; Margolis et al. 2005; McAusland and Costello 2004).

Not all non-native or introduced species are responsible for causing economic damage. A minimum of 4542 species currently existing within the United States originated from outside its borders (United States Congress 1993). This includes important agricultural commodities such as corn, wheat, and rice, as well as cattle, poultry, and other livestock. Additionally, many introduced species can have potential conservation benefits if they provide food for native species, substitute for extinct species in an ecosystem, or act as a catalyst for restoration (Schlaepfer et al. 2011). Both introduced and invasive species are not native to the host environment; however, invasive species are harmful, whether measured economically, environmentally, or as a human health hazard (No EO 1999).

The framework this paper presents will focus on vertebrate invasive species (VIS)—a subset of invasive species that includes bony fish, sharks, rays, amphibians, reptiles, mammals, and birds—to present a method for deconstructing sources of damage. Earlier work suggests VIS invasions may be increasing and are responsible for a sizeable amount of environmental, ecological, and agricultural damages (Vilà et al. 2010). Estimating the direct economic impact and potential future economic impact of VIS is crucial for targeted prevention, management, and control efforts (McNeely 2001; National Invasive Species Council 2001). Prevention of invasions into vulnerable areas necessitates an understanding of the potential economic impacts associated with the establishment of an invasive species. To generate funding to fight an established VIS or to prevent the expansion of a VIS, it is necessary to understand the full range of potential economic impacts.

Existing studies of VIS are principally species-specific, limited geographically, and only examine the direct economic impact to agricultural production (Engeman et al. 2010). Very few studies categorically differentiate VIS damages to examine direct or broader downstream impacts (Shwiff et al. 2017a, b). The goal of invasive species management is to determine biologically effective and economically feasible methods of prevention, control, and damage mitigation. This study aims to provide a general method for delineating the immediate observable *negative* impacts associated with VIS in the United States—not addressed are the potential benefits, such as recreational value. Using examples of commonly discussed problem invasives, we show how different types of VIS cause damage through distinct mechanisms and then present an overview of the feral swine problem as a unique case where we observe a species causing significant damage.

## Framing the economic impacts of VIS

The direct economic damage or harm created by a VIS typically falls into three broad categories: destruction, depredation, and disease transmission. We refer to these classifications of VIS damage as the “Three D’s,” and they represent the core of the damage evaluation framework we propose in this paper.

Destruction encompasses the effects of VIS-related damage to property, equipment, and habitat including any associated “destroyed” or reduced recreational opportunities and is perhaps the broadest category of VIS impact. Examples include damage to statues, golf courses, ecosystems, vehicle collisions, non-consumptive crop damage (e.g. rooting by feral swine), non-consumptive harassment of livestock, lost tourism opportunities, and many others (Campbell and Long 2009; Kaller and Kelso 2006; Engeman et al. 2008; Bevins et al. 2014; Daszak et al. 2000; Depenbusch et al. 2011; Doody et al. 2014; Hartin et al. 2007; Jones et al. 2008; Loss et al. 2013; Shwiff et al. 2010; Yang et al. 2014). Depredation refers specifically to the consumption of crops, livestock, companion animals, or wildlife. Crop and livestock predation has been particularly well-documented, given their clearly defined markets and central role in U.S. agriculture. For example, Pimentel et al. (2005) summarize several reports of crop damage caused by European starlings and estimate that the annual damage is approximately $800 million per year. The final category, disease, refers to mortality or morbidity in humans, companion animals, livestock, or wildlife caused by VIS-associated pathogens (Witmer et al. 2003; Campbell et al. 2008; Hall et al. 2008). This category can be more difficult to quantify but is particularly concerning due to its potential impacts to human health.

Most invasive species have impacts that fall within two of the three categories. Reptiles (e.g., Burmese pythons and brown tree snakes) and aquatic non-native species typically result in negative economic impacts through depredation and environmental destruction, but rarely through disease transmission (Greene et al. 2007; Snow et al. 2007). Some avian species, such as European starlings, can be responsible for damages in the destruction as well as disease categories. Starlings create significant losses through crop destruction, but they also damage property (e.g., statues, bridges, etc.), and are known to be a vector for disease (e.g. fecal contamination of livestock feed) (Shwiff et al. 2012). Crop depredation constitutes the majority of avian damages, while the disease transmission contributes significantly less to the overall impact. Similarly, rodent VIS can also cause damage in all these areas but seem to have a concentrated impact in crop depredation. Feral swine, however, can create significant impacts in all three categories. By far the most significant amount of research has examined the impact of feral swine to crop depredation; however, additional work has provided substantial estimates in other damage categories as well.

Most research results from VIS studies provide primary damage estimates for each of the three D’s, which typically result in secondary economic impacts that in turn effect the broader economy. To have comprehensive and accurate damage estimation, it is necessary to quantify both primary and secondary economic impacts of VIS.

## Methods of valuation: primary and secondary

Primary impacts refer to directly observed economic effects associated with the damage caused by VIS and these primary losses give rise to secondary impacts. Secondary impacts, or indirect economic losses, are multiplier impacts and downstream implications as the direct impact translates through the macroeconomy, including both lost revenue and jobs. For example: the direct economic impacts of diseased livestock are typically characterized by costs associated with morbidity (increased veterinary visits, increased feed, and decreased production) and mortality (the lost value of livestock). The indirect effects, however, include decreased spending in the local economy by the producer as a result of less disposable income as well as jobs lost in the livestock transportation sector as fewer animals need to be moved. Modeling downstream or supply side effects of destruction can in some cases be similar to depredation, especially when examining the impacts of some VIS to crops, however, in many cases, can be categorically different. For example, when VIS harass range cows or calves this may manifest as reduced weight gain which translates to reduced beef in the supply chain. When a beef cow or calf is depredated by VIS this manifests itself as a removal of beef production in the system. The downstream economic implications of these two categories are very different with depredation having considerably greater impacts than destruction. The destruction category also captures many lost or destroyed recreational opportunities which depredation does not and is modeled very differently in an economic sense.

Valuation of primary damage caused by VIS—through destruction, depredation, and disease transmission—is usually accomplished by estimating the value of the loss, repair, or restoration of the affected resource. Market values are commonly used when monetizing the impact to livestock or crops (Engeman et al. 2010; Cummings et al. 2005; Gebhardt et al. 2011). Loss values can be used to estimate the value of things not actively bought and sold in markets and are often used in the case of death related to disease transmission or depredation of non-livestock like companion animals or humans. Destruction is typically valued by using the cost of repairs or restoration (Engeman et al. 2008). Finally, restoration costs, rehabilitation costs, lost recreational opportunities, or non-market values are often used to quantify economic damages to ecosystems and wildlife (Engeman et al. 2004, 2005).

Primary damage tends to be more readily quantifiable as its impacts are immediately observable; however, this damage can be related directly to non-market resources such as ecosystems and therefore putting a precise pecuniary value can be rather ambiguous. Whenever market values are not available, alternative valuation methods are often used to quantify VIS damages. Non-market valuation of wildlife or the recreational value of natural resources can be achieved using survey-based methods such as contingent valuation and travel cost methodology, as well as non-survey methods like benefit-transfer (Loomis and Walsh 1997). While these can provide some insight into the lost economic opportunity arising from VIS damage, they are imprecise as the numbers can be biased due to the subjective choice of methodology and limitations of survey responsiveness.

Primary damages can generate secondary impacts due to economic factors that create linkages to established economic sectors. For example, the primary damage associated with invasive bird damage to dairies is estimated based on the market value of the lost milk. When the milk is removed from the supply chain, additional downstream losses occur to industries linked to milk production (e.g. bottlers and retailers) which would be considered a secondary economic impact (Elser et al. 2019).

The magnitude of secondary damages can be significant due to the multiplier effect of indirect damage. These downstream effects can be observed in all three of the D’s. Regional economic analysis (REA) determines an estimation of secondary impacts associated with VIS to macroeconomic indicators such as revenue, income, and jobs. VIS depredation of sunflower crops (the direct effect) generates measurable secondary impacts such as decreased sunflower oil production (indirect effect). Decreases in sunflower oil production impact the regional economy and can be measured using regional economic models. Macroeconomic changes that arise from decreased sunflower production due to VIS damage can be analyzed using computer software models like the REMI PI + software. REMI is a computer-based simulation model of the US economy that allows modeling at both the national and sub-national scales. This structural economic forecasting model uses a non-survey-based input–output (I–O) table, which models the linkages among industries and households of a regional economy (Shwiff et al. 2015). Using the REMI model, we can generate forecasts that detail behavioral responses to changes in price, production, and other economic factors (Treyz et al. 1991). In other words, REMI can model the impact that changes in the agricultural sector might have on other sectors of the economy and predict changes in employment and income in those sectors. For example, a decrease in sunflower production may result in decreased spending at local restaurants and retail shops, which in turn generates job loss at those businesses. This decreased income among workers then translates into a further decrease in spending. Capturing these ripple effects, or multiplier effects, is vital to understanding the total impact a change in one sector has on the entire regional economy (Miller and Blair 2009).

## Examples of 3 D’s primary damage in the literature

Most often estimates of damage are aggregated across the three categories and studies may report destruction and depredation impacts as a single number. This tends to make these types of studies not replicable and difficult to extend or extrapolate to other areas. In the case of studies that simply itemize damage, we have listed those impacts under the destruction category. In examining the published estimates of economic damage created by invasive species, it is clear that there is a paucity of research in this area. This explains why the most widely cited estimate of the total damage from bird, mammal, reptile, and amphibian invasive species is $39.4 billion annually (Pimentel et al. 2000, 2005). Additionally, Pimentel et al. (2005) estimates the annual control costs are $11.5 million, although feral pig and brown tree snake control costs are the only costs included.

1. VIS damage, excluding feral swine

Marbuah et al. (2014) present a general review of national scale studies of invasive species and their associated damages. The review finds that estimates of economic damage can vary broadly depending on geographic region, duration of study period, and classification of species (vertebrate, invertebrate, plant, etc.). For instance, a 1993 report from the United States Office of Technology Assessment (OTA) (Congress US 1993) reported that economic damages from a group of 79 invasive species—9 of which were invertebrates—over 85 years totaled $185 billion (2016 USD) in the U.S. alone. The same study estimated that terrestrial VIS were responsible for $39.4 billion in economic damage annually (Table 1). As further evidence of the substantial variability in damage estimates, a separate report from USDA-APHIS-Wildlife Services stated that for the federal fiscal years of 1990–1997 annual damage from invasive reptiles, mammals, and birds were $1.2 million, $1.4 million, and $28 million, respectively (Bergman et al. 2002). Yet another report that focused on introduced rats (*Rattus rattus*) and estimated annual damages to be $21.2 million (Pimentel et al. 2005).

**Table 1:** Annual estimates of VIS destruction (United States Congress 1993)

VIS	Annual estimate (in millions)
Wild horses	$5
Mongooses	$50
Rats	$19,000
Cats	$17,000
Dogs	$250
Pigeons	$1100
Starlings	$800
Brown tree snakes	$1

All figures have been adjusted to 2018 USD

The brown tree snake has proven to be an especially pernicious VIS in its ability to cause significant economic damages. On the small island of Guam in the North Pacific, the snake is known to damage property and reduce productivity by causing frequent power outages with an estimated loss of $4.5 million over a seven-year period (Fritts 2002). Especially concerning about the brown tree snake is its capacity to cause pronounced damage in a very small economic and geographic region and thus, its potential to cause enormous losses if it were to spread to a larger economy. Shwiff et al. (2012) used data from the snake’s invasion on Guam, along with survey information from Hawai’i, to estimate the cost of a potential invasion into Hawai’i. Results suggested that total annual damage to the tourism-based economy from such an invasion would be between $593 million and $2.14 billion.

One of the most common forms of damage by VIS is agricultural losses. Invasive bird species like starlings, are common culprits of agricultural depredation as they frequently forage in crop-intensive areas. Recall that the impact of European starling depredation mentioned previously reached $800 million annually (Pimentel et al. 2005). This figure is a reflection of both the population of starlings in the U.S. as well as their ability to inflict crop losses. This estimate is based on losses from grain fields, however starlings have also been found to cause substantial damage to fruit production, such as cherries. Rodent invasives, notably rats, are also capable of creating large economic damages of up to 10% of annual crop harvests or stored grains (Singleton 2003).

Bergman et al. (2002) calculated that between fiscal years 1990–1997, the most frequent requests for assistance for invasive mammals in the U.S. were related to livestock predation by invasive canines. Invasive dogs (*Canis* spp.) were responsible for 20% of the total damage reported to USDA-APHIS-Wildlife Services during that time. The distinction between invasive and introduced is important to keep in mind in this case. Invasive dogs refer to introduced canines that are causing damage. This includes species that may have been introduced as companion animals and have since become feral but does not include native species like wolves (*Canis lupus*) or coyotes (*Canis latrans*). Despite the undeniable impact of VIS depredation, there appears to be less work on vertebrate species than other classifications of invasive species (Marbuah et al. 2014) The paucity of literature on these species identifies an important area for future research.

Published estimates detailing the economic impact of VIS-associated disease transmission are scant. While it is generally known that VIS play an important role in the transmission of transboundary disease between humans, wildlife, and domestic animals, it is difficult to translate that impact into dollar terms. It is estimated that wildlife—some, but not all, of which are VIS—play a role in 79% of the reportable domestic animal diseases and, of those diseases, 40% are zoonotic (Miller et al. 2013). For example, the common pigeon (*Columba livia*) and the European starling are known carriers of dozens of diseases that pose a threat to human and livestock health and safety (Weber 1979). Avian malaria was introduced to Hawai’i by exotic birds kept as companion animals by settlers and is believed to be at least partially responsible for the extinction of at least ten bird species on the island (Lowe et al. 2000) In the continental United States, the invasive nutria (*Myastor coypus*) can be found across the Gulf Coast and can carry tuberculosis and a host of parasites that are hazardous to water supplies and recreation areas (USDA-APHIS-WS 2010).

2. Management and control

The costs of controlling invasive species populations and dispersal is a separate but related area that also has substantial economic impacts. As discussed, VIS are capable of creating pronounced economic damage in addition to being an ecological and environmental threat. Accentuating the issue is that, like many environmental problems, the provision of VIS management is a public good and thus if this provision is left to the private sector it will be allocated inefficiently (Perrings et al. 2002). Accordingly, government agencies and regulators are typically responsible for VIS management. In 2011 alone, the U.S. Department of the Interior spent $100 million on invasive species prevention, early detection, rapid response, management, research, outreach, international cooperation, and habitat restoration (U.S. Fish & Wildlife Service 2012b) Along with the Department of the Interior, the U.S. Fish and Wildlife Service (USFWS) are particularly active in VIS management. For example, the USFWS and its partners have spent $2 million working with 15 trappers to eradicate over 8000 nutria from Maryland’s Blackwater National Wildlife Refuge (U.S. Fish & Wildlife Service 2012a). Other projects include managing Burmese python and other large constrictor snake populations in the southeastern U.S. to protect endangered species such as the Key Largo woodrat (*Neotama floridana small*) and wood stork (*Mycteria americana*). Since 2005 the USFWS and its partners have spent over $6 million on these programs and prevented the extinction of several species (US Fish & Wildlife Service 2012b, c). The United States Geological Survey (USGS) has also devoted significant resources to VIS research and control. Annually, the USGS requires $4 million in research costs in addition to normal operating costs for management of Guam’s National Wildlife Refuge and military environmental programs (USGS 2013). Although control costs do not factor into the “three Ds” that are central to the framework constructed in this paper, they still represent an important component of the economic issues surrounding VIS. Allocation of public funds to VIS management illustrates the importance that the public sector attributes to combatting the negative impacts of these species.

## Feral swine

**Fig. 1:**
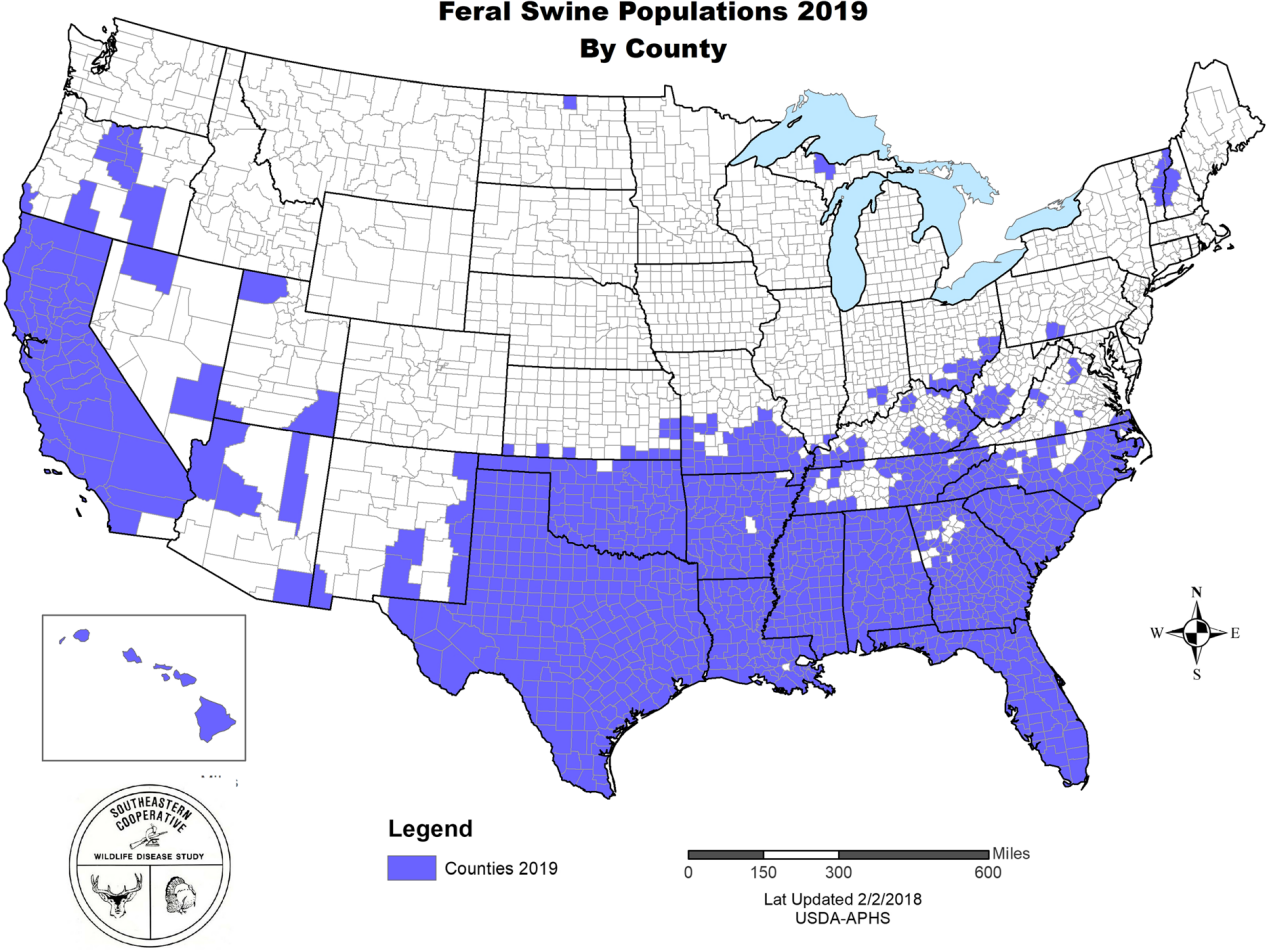
2019 Feral swine populations in the United States, by County (National Feral Swine Damage Manegement Program 2019)

Feral swine have experienced significant range expansion over the past 30 years, in part due to translocation by hunters who desire a local hunting opportunity (Bevins et al. 2014; Acevedo et al. 2006; Saito et al. 2012; Spencer and Hampton 2005). Feral swine have existed in pockets of the southeastern U.S., California, and Hawai’i for nearly five-hundred years, and recent trends indicate a general northward expansion of populations (Anderson et al. 2016). This expansion has also increased conflicts with agriculture and humans in the areas where feral swine exist, emphasizing the need for assessing the costs and benefits associated with the presence of feral swine in different localities (Campbell and Long 2009; Bevins et al. 2014; Anderson et al. 2016; Campbell et al. 2013; Engeman et al. 2013; Higginbotham 2013; Higginbotham et al. 2008; Mengak 2012; Ober et al. 2011; Siemann et al. 2009). In addition, there has been considerable research conducted on the increasing potential for management conflicts stemming from feral swine expansion (Honda and Kawauchi 2011; Koichi et al. 2013; Warner and Kinslow 2013; Weeks and Packard 2009) (Fig. 1).

1. Destruction

The most commonly cited publication regarding feral swine damage reports an estimated annual impact of $800 million ($1.03 billion 2018 USD) resulting from crop and environmental damage (Pimentel et al. 2005). One of the more comprehensive studies was a survey of 11 states (Alabama, Arkansas, California, Florida, Georgia, Louisiana, Mississippi, Missouri, North Carolina, South Carolina and Texas) distributed by the USDA National Agricultural Statistical Service in the summer of 2015 (Anderson et al. 2016). The survey sampled producers of corn (*Zea mays*), soybeans (*Glycine max*), wheat (*Triticum*), rice (*Oryza sativa*), peanuts (*Arachis hypogaea*), and sorghum (*Sorghum bicolor*) in the 11-state region. They extrapolated crop damage estimates to the state level in 10 states with reportable damage yields to determine an estimated annual crop loss of $190 million. Researchers in Georgia found that 9.7 million acres in that state suffered $57 million in crop destruction in 2011 (Mengak 2012). According to USDA NASS (n.d.), this area is responsible for approximately one percent of total U.S. crop sales.

In addition to crop damage, feral swine can destroy natural habitats and even personal property. In South Carolina, one study examined the property damage associated with vehicular collisions involving feral swine. The study considered 179 collisions involving feral swine and found an average damage estimate of $1173 per collision. Seward et al. (2004) emphasized the ecological and environmental damages associated with feral swine including erosion damage and the predation of endangered or threatened animal species such as marine turtles and their nests.

Table 2 summarizes significant estimates of damage by feral swine found in the literature. The base year of 2012 was chosen to put these figures on par with the most recent USDA Census of Agriculture and was adjusted for inflation to 2016 prices. When possible, the data was converted into annualized costs. Given the great variety in the existing research, the comparison of costs across differing localities, crops, and time scales is problematic. For example, Higginbotham et al. (2008) found feral swine cause $58 million/year in damage to the whole of Texas agriculture, an area of 59 million acres. However, Mengak (2012) reported a similar $58 million/year for crop damages to 9.7 million acres in Georgia, only part of which was agricultural land. It is difficult to reconcile that such different sized areas experienced similar levels of reported damage, highlighting the inherent difficulty in estimating agricultural damages from feral swine. The numbers in parentheses next to each state represent the number of studies which provided estimates.

**Table 2:** Estimates of feral swine destruction (Beach 1993; Westenbroek 2011; Hall 2012; Tolleson et al. 1995; Anderson et al. 2016; Ober et al. 2011; Mengak 2012; USDA APHIS WS 2010; Frederick 1998; Mayer and Johns 2011; Adams et al. 2005; Higginbotham et al. 2008; Engeman et al. 2003, 2004; Sweitzer and McCann 2007)

Geographical area	Crops- single incidents description	Estimates
Texas (7)	Peanuts	$64,803
New York (4)	Corn	$15,157
New York (4)	Corn, Apples, and Strawberries	$25,000

Geographical area	Crops—annual aggregates description	Estimates
Texas (1)	Peanuts	$225,518/year
Texas (1)	N/A	$15,492–$464,765/year
Texas (17)	Corn, Soybeans, Wheat, Rice, Sorghum, Peanuts	$89,817,000/year
Alabama (17)	Corn, Soybeans Wheat, Rice, Peanuts	$21,322,000/year
Arkansas (17)	Corn, Soybeans Wheat, Rice, Peanuts	$19,575,000/year
Florida (17)	Corn, Soybeans Wheat, Rice, Peanuts	$5,985,000/year
North Florida (3)	Corn, Cotton, Peanuts, and Soybeans	$1,921,224/year
Georgia (6)	Reported Crops- Mengak (2012, p. 13) SW Extension District	$58,180,000/year
Georgia (17)	Corn, Soybeans Wheat, Rice, Peanuts	$5,150,000/year
Louisiana (17)	Corn, Soybeans Wheat, Rice, Peanuts	$15,670,000/year
Mississippi (17)	Corn, Soybeans Wheat, Rice, Peanuts	$18,518,000/year
Missouri (17)	Corn, Soybeans Wheat, Rice, Peanuts	$485,000/year
North Carolina (17)	Corn, Soybeans Wheat, Rice, Peanuts	$4,684,000/year
South Carolina (17)	Corn, Soybeans Wheat, Rice, Peanuts	$8,747,000/year

Geographical area	Property description	Estimates
New York (12)	Two lawns	$421 each
Georgia (6)	Property Damage in SW extension district	$24,500,000/year
California (8)	31 Residential Properties and 1 Golf Course	$93,652/year
Nationwide (13)	Avg. Property Damage from feral swine—vehicle collisions	$1197/car

Geographical area	Total uncategorized description	Estimates
Texas (9)	“Economic Loss Since Feral Swine Appeared on the Respondent’s Property” (Adams et al. 2005, p. 1316)	$3,225,796
Texas (10)	Cost to Texas Agriculture	$57,580,650/year
Texas (10)	Repairing Damage and Control	$7,751,242/year
California (8)	Total Reported Damage to Hay, Forage, Ponds, Lawns, Drainage, Orchards, Vineyards, Irrigation, Livestock, Crops, Trees, Fruits, and Nuts	$2,634,343/year

Geographical area	Environmental description	Estimates
Florida (14)	Value of damaged area of Savannas Preserve State Park	$1,545,717–$5,036,456
Florida (15)	Damage to three FL state parks at the end of the study period	$6652–$28,384/ha
California (16)	Damage and Control	$400,169/year

All figures have been adjusted to 2018 USD

Feral swine have inflicted considerable environmental costs mainly through rooting, grubbing, or wallowing (Engeman et al. 2004; Seward et al. 2004). Seward et al. (2004) attribute the decline of twenty-two species of plants and four species of amphibians to feral swine. In addition, damage to marshes and parks by feral swine has been noted (Pimentel et al. 2005; Engeman et al. 2004; Engeman et al. 2003). Feral swine also damage an unknown amount of priceless archaeological sites (Engeman et al. 2013) and were found to “dominate the disturbance regime” of the Northern California Coast Range Preserve (NCCRP) (Kotanen 1995). Table 2 also contains estimates of environmental damage inflicted by feral swine.

2. Depredation

There is very little quantitative data published about the predatory behavior of feral swine; however, what is lacking in quantitative data is offset by what is known in qualitative terms. Using DNA analysis of stomach contents, Robeson et al. (2018) identified a diversely omnivorous diet including animal and plant matter unique to locations and environmental conditions highlighting the opportunistic depredatory hazard posed by the spread of feral swine. This section will review what information is available about depredation by feral swine. Surveys, qualitative reports describing feral swine attacks, and anecdotal evidence are available from several sources. Survey respondents were individuals concerned about, or those who had experienced, an attack, livestock depredation, and damage or injury to pets (Mengak 2012; Sweitzer and McCann 2007; Barrett and Pine 1981; Rollins 1998).

Feral swine regularly consume a multitude of crops, including sugar cane, wheat, peanuts, grain sorghum, rice, and corn. Jerrolds et al. (2014) conducted a survey of agricultural groups and resource managers in Tennessee and found that 94% of counties had swine populations and the majority of complaints were related to crop and pasture damage. To provide an understanding of the impact of feral swine crop depredation alone, there is some anecdotal evidence of considerable losses realized in New York. Hall (2012) discusses a farm in Clinton County suffering $25,000 in losses from corn, apple, and strawberry depredation. Westenbroek (2011) discusses a farm in Delaware County that lost $14,850 to feral swine consumption of corn fields.

Of particular importance to agriculture is the fact that feral swine are known to prey on livestock. Primarily, feral swine prey on sheep (*Ovis aries*) and goats (*Capra hircus*), but have been known to feed on larger animals such as cows (*Bos taurus*) and other exotic game species leading to substantial economic loss (Seward et al. 2004; Frederick 1998). Christie et al. (2014) report communications claiming feral swine are preying on calves in Kern County, California. Feral swine presence pressures sheep herds, leading to increased abortion rates of lambs at such frequency that a relationship can be derived between feral swine densities and lamb survival (Choquenot et al. 1997). Seward et al. (2004) report the characteristics of feral swine predation and it is believed that feral swine kills may be mistakenly reported as coyote kills leading to a possible under-reporting of feral swine depredation. Anecdotally, a rancher in Texas experienced a 15–20% reduction in goat kid production on property where feral swine reside (Beck 1999). Barrios-Garcia and Ballari (2012) report that around 30% of feral swine diet consists of animal matter depending on ecosystem and season. At a value of about $110,669 in 2012 USD 1243 head of sheep and goats were documented as lost to feral swine by Texas authorities in 1990 (Rollins 1998). In 1991, 1473 sheep, goats, and exotic game animals were reportedly killed by feral swine in Texas and California (Barrett and Birmingham 1994). Seward et al. (2004) report that feral swine cause greater than $1.2 million in goat losses annually. Attacks on humans and endangered species are detailed in court cases and peer reviewed literature. Love (2013) details the case of an inmate on a work crew who was attacked by a feral swine. Mayer (2013) found that up to 15% of reported feral swine attacks on humans are fatal. Furthermore, feral swine are also known to feed on threatened and endangered species (Bevins et al. 2014; Bengsen et al. 2014) as well as cause severe damage to vulnerable wetlands and biodiversity hotspots such as Florida’s steephead ravines (Engeman, et al. 2007). Engeman et al. (2016) found that management of feral swine populations in Cayo Costa along Florida’s west coast led to a significant rebound in threatened sea turtle and shorebird populations when compared to non-treated ecosystems on North Captiva which has seen a dramatic decline in these threatened species.

Without a larger body of quantitative work, it is difficult to assess the threat feral swine pose to livestock. The available qualitative research reveals that feral swine depredation is a real problem to a number of different agricultural producers. Further research and more robust data collection will be necessary in order to effectively quantify feral swine depredation costs.

3. Disease

Feral swine are a potential reservoir of both zoonotic and non-zoonotic diseases that could impact the U.S. economy through a number of channels (Miller et al. 2017). Of the 42 serious pathogens with a wildlife component reported by Miller et al. (2013), feral swine are explicitly involved in seven. Feral swine pose a threat as a potential vector for new forms of the influenza virus as they have the required receptors for both avian and human strains of the virus and this provides an opportunity for the viruses to reassort (Hall et al. 2008). Survey respondents indicated concern or experience with feral swine spreading disease to livestock or acting as a potential disease reservoir (Barrett and Pine 1981; Rollins 1998). They have also been known to carry pathogens that pose a danger to humans (Bengsen et al. 2014).

While the disease threat posed by feral swine is clearly recognized within the literature, it has thus far been difficult to accurately model the spread of a disease outbreak vectored by feral swine. The current iteration of disease transmission models is largely focused on the spread of a single disease between a limited number of species (e.g. Ward et al. 2007, 2009). However, the complexity of the feral swine problem requires a model flexible enough to extend into the transmission of multiple pathogens across multiple species, with virtually unrestricted pathways of introduction.

The cost of one outbreak of foot-and-mouth disease (FMD) in the United States involving feral swine is estimated to range from $7.5 million to $5.8 billion for a single state (Cozzens 2010; Cozzens et al. 2010). Feral swine have been identified as an important reservoir for other transboundary animal diseases such as classical and African swine fever viruses and also for production diseases such as porcine reproductive and respiratory syndrome (Müller et al. 2011; Jori and Bastos 2009). In addition to these domestic swine diseases, there is increasing concern over the potential losses in cattle and other domestic livestock associated with transmission of pathogens such as pseudorabies virus (Aujeszky’s disease) (Bitsch 1975; Crandell 1982; Hagemoser et al. 1978).

Research on pathogen transmission between wildlife, specifically feral swine, and livestock has been progressing. Pineda-Krch et al. (2010) developed a disease transmission model, which included elements of both space and randomness, to simulate the spread and control of FMD among beef and dairy herds and feral swine in California. Results show that introduction of FMD from feral swine to livestock could result in a large and rapidly moving outbreak. Tested containment strategies showed potential to reduce the size and duration of the outbreaks.

Ward et al. (2009, 2007) built a disease spread model that explicitly modeled the potential for FMD spread between domestic cattle, feral swine, and white-tailed deer in Texas. The model considered geographic relationships between the species and found that interspecies contact, distribution of affected animals, and densities of the species were important in determining the extent of the outbreak (Ward et al. 2007, 2009).

The challenge beyond modeling an outbreak is valuing the potential damage to the agricultural sector and the economy as a whole. The potential for damage through commercial livestock production is related to the number of exposed head of livestock. Total U.S. livestock production totaled $90 billion in 2012 (USDA NASS n.d.) with $5 billion in beef exports (USDA-ERS 2013), and $6.3 billion in pork exports (MEF and U 2014). Exports account for almost 13% of total beef production and 27% of pork production (MEF and U 2013). Even limited outbreaks can be exceptionally costly, due to the potential for international banning of U.S. imports with price effects for the entire U.S. herd of the affected species. Coffey et al. (2005) estimate that the single reported case of bovine spongiform encephalopathy (BSE) in December 2003 cost the U.S. beef industry between $3.9 and $5.7 billion from lost exports in 2004.

Some of the only studies to date that have explicitly focused on feral swine in an economic context are Cozzens (2010) and Cozzens et al. (2010), which modeled the economic impact of a feral swine introduced FMD infection to domestic livestock. Cozzens (2010) found that potential producer losses in Kansas due to feral swine transmission of FMD to domestic livestock could be as much as $6.1 billion. Total economic impact for the occurrence of FMD in livestock as a result of exposure to infected feral swine in Missouri was estimated at $12.6 million (Cozzens et al. 2010).

There are also concerns regarding contamination of the human food supply by feral swine. Disease events can generate economy-wide impacts across both consumers and producers, as illustrated through the deadly September 2006 *E. coli* O157:H7 outbreak in which feral swine were implicated in having contaminated spinach (Kreith 2007). Consumer expenditures on leafy greens declined by $69 million and spinach producers lost an estimated $234.4 million as lettuce and similar produce were substituted for spinach (Arnade et al. 2009).

In addition to these direct concerns regarding the food supply, there is also the general threat of feral swine acting as a vector of disease. Feral swine are a known vector of influenza, and initially the 2009 outbreak of H1N1 influenza was called “swine flu” by authorities. This mislabeling led to substantial negative consumer response, even though Attavanich et al. (2011) determined that pork remained safe to consume throughout the entire event. It was estimated that agricultural sector losses of $159 million were associated with the “swine flu” media coverage. Not only do feral swine have the potential for disease transfer through the food supply, but it has also been seen that they pose a zoonotic risk to food processors. Pederson et al. (2017a, b) found antibodies to multiple zoonotic pathogens including *Leptospira* in almost half of feral swine tissue samples from Texan abattoirs. Employees in abattoirs that slaughter swine are at significant risk of exposure to zoonotic illness; cases of brucellosis and leptospirosis have been reported among employees working on processing plant kill floors in many states with feral swine (Campagnolo et al. 2000; Pedersen et al. 2015; Trout et al. 1995).

The ability to directly study and measure the impacts of a multi-species, multi-pathogen feral swine induced epidemic is still beyond the scope of currently available models. However, evaluation of the costs associated with FMD and BSE outbreaks between feral swine and species such as cattle or deer show the damaging potential of even small-scale disease transmission events. In addition, there are substantial costs stemming from both real and perceived food safety threats. While the full magnitude of the disease impacts is not currently known, it is clear from the available evidence that the disease potential posed by feral swine is a legitimate threat to the U.S. agricultural sector.

4. Management and control costs

Given our discussion of the kinds of damage feral swine cause, it is no surprise that considerable effort and resources have been devoted to the control and management of feral swine populations. There is substantial interest in an accurate measure of feral swine management costs, especially as a point of comparison with the damages incurred. The need to control this population implies a need for better information regarding the feral swine density and distribution. The feral swine population in Texas has been estimated at 2 million animals (Higginbotham et al. 2008). Current nationwide population estimates exceed 6 million feral swine (Higginbotham et al. 2008; Pimentel 2007; USDA-APHIS-WS 2013). However, census data is extremely difficult, and few studies have generated a reliable national population estimate for the feral swine population.

It is known that feral swine are incredibly prolific; Hanson et al. (2009) found that feral swine are capable of speeding up their reproductive cycles under pressure, and Bengsen et al. (2014) found that feral swine reproduction rates can increase as their population decreases below the local carrying capacity. All of these factors combine to create unique and costly challenges in the management and control of feral swine. This is borne out by the research of Saunders and Bryant (1988) who found an asymptotic relationship between control efforts and control success. In fact, studies have shown that lethal control efforts must result in mortality rates ranging between sixty and eighty percent in order to impact the ability of feral swine to maintain their population (Barrett and Pine 1981; Ward et al. 2009; Kreith 2007; Hone and Pedersen 1980). The cost estimates for feral swine and management are presented in Table 3.

**Table 3:** Costs associated with the control of feral swine (Engeman et al. 2004; Sweitzer and McCann 2007; Kreith 2007; Hone and Pedersen 1980; Saunders and Bryant 1988

Geographical area	Description	Estimates
California (16)	Feral Swine Related Management Costs Incurred by Natural Areas in California	$4.49 m/year
California (16)	Feral Swine Eradication Efforts During Study Period (3 years)	$4.07 m/year
California (16)	Construction and Maintenance of Exclusion Fence at Pinnacles National Monument (~ 20 years.)	$61,104/km
California (18)	Construction of Exclusion Fence at Pinnacles National Monument	$1,958,251
California (18)	Eradication Efforts at Pinnacles National Monument	$1,101,843
California (18)	Maintenance of Exclusion Fence at Pinnacles National Monument	$71,803/year
Florida (14)	Average Removal Cost	$43.08/head
Texas (11)	Average Removal Cost	$72.83/head
Australia (24)	Average Removal Cost	$95.84/head
Australia (25)	Average Removal Cost	$17.28/head

All figures have been adjusted to 2018 USD

Methods of feral swine control deemed acceptable differ by stakeholder groups. Koichi et al. (2013) found that acceptability of management practices was influenced by stakeholder group identification (e.g. residents vs. tourists), awareness of a feral swine problem, and social factors influenced the views of each of the stakeholders. For example, Weeks and Packard (2009) found that feral swine are so well established in the local culture around a National Park in Texas that residents do not consider them non-native. Control efforts are met with considerable resistance, especially when professional hunters are hired. Furthermore, Warner and Kinslow (2013) found feral swine control efforts conducted by “outsiders” in Hawai’i (e.g. U.S. federal agencies) without public consent have been met with strong public opposition. These conflicting views of different stakeholders are but one of the primary hurdles to engaging in effective feral swine management. Recent research in the field of human dimensions have shown many factors can slow the progress of invasive species management. In a 2017 study of Tennessee landowners in counties with feral swine, only 49% indicated they would consider allowing government officials on their property to control feral swine (31% were unsure and 21% were against the idea) (Caplenor et al. 2017). Similar recent studies have shown a public resistance to certain methods of swine elimination—particularly strong opposition to the cost-effective use of toxicants (Harper 2016; Harper et al. 2016)—as well as public attitudes amongst certain demographics reluctant to support government involvement in controlling feral swine (Caplenor et al. 2017).

## Discussion

We have identified a general framework that can be utilized for categorizing VIS damage divided into three main categories: destruction, depredation, and disease. These three categories represent the most important economic concerns associated with VIS, yet most current literature only focuses on one or two categories. Within this framework, we have sought to provide a comprehensive review of the literature available on the VIS impacts within the U.S.

Our review of the literature encompassing the costs associated with VIS reveals an incomplete and biased understanding of the economic damages and control costs. For example, rats and cats are two of the most negative VIS in terms of monetary impacts; however, they are ubiquitous in the U.S. and in some instances are not considered invasive. Burmese pythons in comparison, are considered to be alarmingly invasive, but are significantly less detrimental and limited to a relatively small geographic region. Currently, the literature does not contain adequate large-scale estimates of damage and what estimates there are feature inappropriate methods of aggregation. In addition, many of these estimates come from a single source: Pimentel et al. (2005). Such limited evaluations highlight the need for more studies that produce comparable results that can be replicated.

This review highlights some important features of VIS damage. For example, while destruction has been the most thoroughly studied area of VIS damage, its estimates vary in scope and approach, making cross-study comparison difficult. Further, when estimates are not comparable, any attempt to aggregate this information to a national level is nearly impossible. Geographic scales range from as small as a single farm to as large as the entire state of Texas. Additionally, studies commonly emphasize different types of destruction (property, environmental, etc.). When studies focus on one geographical region or type of destruction, it may not always be appropriate to extrapolate that information to a larger scale.

An important aspect of VIS identified by this study is that feral swine damage is substantial, pervasive, and poised to become the most significant contributor to damage of all VIS. In this review, the damage caused by feral swine falls under all categories (i.e., destruction, depredation, and disease transmission) but the largest portion of damage occurs mainly through destruction. Much of the destruction created by other VIS discussed is limited by crop or region; however, feral swine do not seem to face that same limitation. Feral swine can cause extensive harm to numerous agricultural, natural, and anthropogenic resources, whereas other VIS may significantly harm crops but cause considerably less damage to other resources.

The second damage category, depredation, suffers from a peculiar problem in that VIS frequently attack or consume agricultural and livestock products but it is difficult to verify the data. Research is expanding in the area that would allow for more accurate identification of the offending species and allow for a more accurate estimation of VIS impacts. For example, genetic testing of material left behind by the offending animal has provided a means by which to verify the species involved in a depredation event (Williams and Johnston 2004). Unmanned aerial systems are also providing a means by which crop depredation events can be systematically captured and accurately accounted for in real time. Drone data footage can be downloaded and run through machine learning algorithms that have been trained to identify crop depredation events that are linked to a particular species. This research likely represents the future of estimating the economic impact of wildlife species in general and not just VIS. Additionally, qualitative information is plentiful and may provide researchers with signals that can identify VIS predation. In terms of depredation, this review did not illustrate a clear VIS leader in this category. Many of the VIS examined here can consume crops but are limited to certain types of crops and very few depredate on livestock.

Disease transmission is arguably the most difficult category of damage to measure but potentially the most important to be addressed and the least researched. As the COVID-19 pandemic has illustrated, the potential for disease spread from wildlife to other species like humans can have substantial economic impacts. Many of the VIS examined in this review can carry and spread diseases and a few studies have actually documented the potential economic impact of those diseases. The literature review conducted here yielded studies that projected the potential economic impacts of disease spread and did not provide an actual accounting of economic impact related to a disease spread event related to a particular VIS. Examining the potential diseases that VIS can carry and transmit to livestock, humans, and wildlife reveals a clear leader for future impacts. Feral swine can act as a host for more OIE reportable List A diseases than any other VIS. Some of these diseases, for example: FMD, can impact a diverse group of livestock and have implications for trade restrictions causing immediate and significant economic losses. The potential impacts for international trade and threat to human health have made disease a common subject within the economics of invasive species literature (Margolis et al. 2005; McAusland and Costello 2004; Zhao et al. 2006). This highlights one of the largest knowledge gaps identified by this review in that there is a lack of research regarding the potential economic impact of diseases spread by all VIS, but especially feral swine. Most studies examining the economic impact of diseases like FMD utilize an epidemiological disease spread model to simulate the spread from a particular location or farm type but do not specify a feral swine component (the one exception is Cozzens et al. (2010)). For instance, many studies concerning VIS lack a discussion of the potential trade implications of disease transmission from VIS to livestock. This is an incredibly important aspect of the economic impact of VIS as indicated by the clear sanitary and phytosanitary measures enacted by the World Trade Organization in the Uruguay round of world trade negotiations (World Trade Organization 1994).

Understanding the economic impacts associated with VIS is crucial. With so many characteristic differences among VIS, it is important to weigh the economic impact as a means to objectively evaluate the damage created by each. In this way, a meaningful management plan can be developed that addresses the most damaging of VIS rather than potentially the most unattractive or frightening. To do this, damages must be categorized in the framework described here to make them comparable across regions and species. The adoption of a standard approach to damage estimation would contribute to the goal of generating national level estimates and forecasts of VIS impacts and potential damages. These estimates could be used as vital inputs to more sophisticated models, such as regional economic models, and provide useful insights to inform policy decisions on VIS management.

## References

[CR1] Acevedo P, Escudero MA, Muńoz R, Gortázar C (2006). Factors affecting wild boar abundance across an environmental gradient in Spain. Acta Theriol.

[CR2] Adams CE, Higginbotham BJ, Rollins D, Taylor RB, Skiles R, Mapston M, Turman S (2005). Regional perspectives and opportunities for feral hog management in Texas. Wildl Soc Bull.

[CR3] Anderson AM, Slootmaker C, Harper E, Holderieath J, Shwiff SA (2016). Economic estimates of feral swine damage and control in 11 US states. Crop Prot.

[CR4] Arnade C, Calvin L, Kuchler F (2009). Consumer response to a food safety shock: the 2006 food-borne illness outbreak of *E. coli* O157: H7 linked to spinach. Rev Agric Econ.

[CR5] Attavanich W, McCarl BA, Bessler D (2011). The effect of H1N1 (swine flu) media coverage on agricultural commodity markets. Appl Econ Perspect Policy.

[CR6] Barrett RH, Birmingham GH (1994) Wild pigs. The handbook: prevention and control of wildlife damage 51

[CR7] Barrett RH, Pine DS (1981). History and status of wild pigs, *Sus scrofa*, in San Benito County.

[CR8] Barrios-Garcia MN, Ballari SA (2012). Impact of wild boar (*Sus scrofa*) in its introduced and native range: a review. Biol Invasions.

[CR9] Beach R (1993) Depredation problems involving feral hogs. Feral swine: a compendium for resource managers. Texas Agricultural Extension Service, Kerrville, TX, USA 67–75. http://agrilife.org/texnatwildlife/feral-hogs/depredationproblems-involving-feral-hogs/. Accessed 24 May 2016

[CR10] Beck R (1999) Eden-Texas. In: First national feral swine conference. 23, 6/2–6/3/1999. Ft. Worth, Texas

[CR11] Bengsen AJ, Gentle MN, Mitchell JL, Pearson HE, Saunders GR (2014). Impacts and management of wild pigs *Sus scrofa* in Australia. Mamm Rev.

[CR12] Bergman D, Chandler M, Locklear A (2002) The economic impact of invasive species to wildlife services’ cooperators 169–178

[CR13] Bevins SN, Pedersen K, Lutman MW, Gidlewski T, Deliberto TJ (2014). Consequences associated with the recent range expansion of nonnative feral swine. Bioscience.

[CR14] Bitsch V (1975). A study of outbreaks of Aujesky’s disease in cattle I. Virol and epidemiol find. Acta Vet Scand.

[CR16] Campagnolo ER, Warwick MC, Marx HL (2000). Analysis of the 1998 outbreak of leptospirosis in Missouri in humans exposed to infected swine. J Am Vet Med Assoc.

[CR17] Campbell TA, Long DB (2009). Feral swine damage and damage management in forested ecosystems. For Ecol Manag.

[CR18] Campbell TA, DeYoung RW, Wehland EM, Grassman LI, Long DB, Delgado-Acevedo J (2008). Feral swine exposure to selected viral and bacterial pathogens in southern Texas. J Swine Health Prod.

[CR19] Campbell TA, Foster JA, Bodenchuk MJ, Eisemann JD, Staples L, Lapidge SJ (2013). Effectiveness and target-specificity of a novel design of food dispenser to deliver a toxin to feral swine in the United States. Int J Pest Manag.

[CR20] Caplenor CA, Poudyal NC, Muller LI, Yoest C (2017). Assessing landowners’ attitudes toward wild hogs and support for control options. J Environ Manag.

[CR21] Choquenot D, Lukins B, Curran G (1997). Assessing lamb predation by feral pigs in Australia’s semi-arid rangelands. J Appl Ecol.

[CR22] Christie J, DeMarco E, Hiroyasu E, Kreger A, Ludington M (2014) Wild pig management on Tejon Ranch. Bren School Group Project report to the Tejon Ranch Conservancy

[CR23] Coffey B, Mintert J, Fox JA, Schroeder TC, Valentin L (2005) The economic impact of BSE on the US beef industry: product value losses, regulatory costs, and consumer reactions

[CR24] Congress US. Office of Technology Assessment (OTA) (1993). Harmful non-indigenous species in the United States.

[CR25] Cozzens TW (2010) Economic impact of feral swine transmitting foot-and-mouth disease to livestock in Kansas. Dissertation, Colorado State University

[CR26] Cozzens T, Gebhardt K, Shwiff S, Lutman M, Pedersen K, Swafford S (2010) Modeling the economic impact of feral swine-transmitted foot-and-mouth disease: a case study from Missouri 308–311

[CR27] Crandell RA (1982). Pseudorabies (Aujeszky’s disease). The Veterinary clinics of North America. Large Anim Pract.

[CR28] Cummings J, Shwiff SA, Tupper S (2005) Economic impacts of blackbird damage to the rice industry. In: Nolte DL, Fagerstone KA (eds) Proceedings of the 11th wildlife damage management conference, vol 11, pp 317–322

[CR29] Daszak P, Cunningham AA, Hyatt AD (2000). Emerging infectious diseases of wildlife: threats to biodiversity and human health. Science.

[CR30] Depenbusch BE, Drouillard JS, Lee CD (2011). Feed depredation by European starlings in a Kansas feedlot. Hum-Wildl Interact.

[CR31] Doody JS, Mayes P, Clulow S (2014). Impacts of the invasive cane toad on aquatic reptiles in a highly modified ecosystem: the importance of replicating impact studies. Biol Invasions.

[CR200] Elser JL, Adams Progar AL, Steensma KM, Caskin TP, Kerr SR, Shwiff SA (2019). Economic and livestock health impacts of birds on dairies: evidence from a survey of Washington dairy operators. Plos one.

[CR32] Engeman RM, Smith HT, Shwiff SA, Constantin B, Woolard J, Nelson M, Griffin D (2003). Prevalence and economic value of feral swine damage to native habitat in three Floridastate parks. Environ Conserv.

[CR33] Engeman RM, Smith HT, Severson R, Severson MA, Shwiff SA, Constantin B, Griffin D (2004). The amount and economic cost of feral swine damage to the last remnant of a basin marsh system in Florida. J Nat Conserv.

[CR34] Engeman RM, Martin RE, Smith HT (2005). Dramatic reduction in predation on marine turtle nests through improved predator monitoring and management. Oryx.

[CR35] Engeman RM, Stevens A, Allen J, Dunlap J, Daniel M, Teague D, Constantin B (2007). Feral swine management for conservation of an imperiled wetland habitat: Florida’s vanishing seepage slopes. Biol Conserv.

[CR36] Engeman RM, Duquesnel JA, Cowan EM, Smith HT, Shwiff SA, Karlin M (2008). Assessing boat damage to seagrass bed habitat in a Florida park from a bioeconomics perspective. J Coast Res.

[CR37] Engeman RM, Laborde JE, Constantin BU, Shwiff SA, Hall P, Duffiney A, Luciano F (2010). The economic impacts to commercial farms from invasive monkeys in Puerto Rico. Crop Prot.

[CR38] Engeman RM, Couturier KJ, Felix RK, Avery ML (2013). Feral swine disturbance at important archaeological sites. Environ Sci Pollut Res.

[CR39] Engeman RM, Addison D, Griffin JC (2016). Defending against disparate marine turtle nest predators: nesting success benefits from eradicating invasive feral swine and caging nests from raccoons. Oryx.

[CR40] Frederick JM (1998) Overview of wild pig damage in California. 18th Vertebrate pest conference, University of California Davis, pp 82–86

[CR41] Fritts TH (2002). Economic costs of electrical system instability and power outages caused by snakes on the island of Guam. Int Biodeterior Biodegradation.

[CR42] Gebhardt K, Anderson AM, Kirkpatrick K, Shwiff SA (2011). A review and synthesis of bird and rodent damage to select California Crops. Crop Prot.

[CR43] Greene DU, Potts JM, Duquesnel JG, Snow RW (2007). Geographic distribution: python molurus bivittatus (Burmese python). Herpetol Rev.

[CR44] Hagemoser WA, Hill HT, Moss EW (1978). Nonfatal pseudorabies in cattle. J Am Vet Med Assoc.

[CR45] Hall W (2012). Wayne’s world: many folks despise them but feral hogs are smart.

[CR46] Hall JS, Minnis RB, Campbell TA, Barras S (2008). Influenza exposure in United States feral swine populations. J Wildl Dis.

[CR47] Hanson LB, Mitchell MS, Grand JB, Jolley DB, Sparklin BD, Ditchkoff SS (2009). Effect of experimental manipulation on survival and recruitment of feral pigs. Wildl Res.

[CR48] Harper EE (2016) Factors predicting feral swine management preferences and willingness to pay. Dissertation Colorado State University

[CR49] Harper EE, Miller CA, Vaske JJ, Mengak MT, Bruno S (2016). Stakeholder attitudes and beliefs toward wild pigs in Georgia and Illinois. Wildl Soc Bull.

[CR50] Hartin RE, Ryan MR, Campbell TA (2007). Distribution and disease prevalence of feral hogs in Missouri. Hum-Wildl Confl.

[CR51] Higginbotham B (2013) Wild pig damage abatement education and applied research activities. Overton, TX: Texas A&M AgriLife Research and Extension. http://feralhogs.tamu.edu/files/2013/06/WildPigDamageAbatementEducationAppliedResearchActivites.pdf

[CR52] Higginbotham B, Clary G, Hysmith L, Bodenchuk M (2008) Statewide feral hog abatement pilot project, 2006–2007

[CR53] Honda T, Kawauchi N (2011). Methods for constructing a wild boar relative-density map to resolve human-wild boar conflicts. Mamm Study.

[CR54] Hone J, Pedersen H (1980) Changes in a feral pig population after poisoning. In: Proceedings of the 9th vertebrate pest conference (1980) p 15. http://digitalcommons.unl.edu/vpc9

[CR55] Jerrolds WR, Pelren EC, Darroch BA, Anderson RG (2014). A survey to estimate population distribution of and damage caused by feral swine in Tennessee. J Southeast Assoc Fish Wildl Agencies.

[CR56] Jones KE, Patel NG, Levy MA (2008). Global trends in emerging infectious diseases. Nature.

[CR57] Jori F, Bastos AD (2009). Role of wild suids in the epidemiology of African swine fever. EcoHealth.

[CR58] Julia R, Holland DW, Guenthner J (2007). Assessing the economic impact of invasive species: the case of yellow starthistle (Centaurea solsitialis L.) in the rangelands of Idaho, USA. J Environ Manag.

[CR59] Kaller MD, Kelso WE (2006). Swine activity alters invertebrate and microbial communities in a coastal plain watershed. Am Midl Nat.

[CR60] Koichi K, Cottrell A, Sangha KK, Gordon IJ (2013). What determines the acceptability of wildlife control methods? A case of feral pig management in the wet tropics world heritage area, Australia. Hum Dimens Wildl.

[CR61] Kotanen PM (1995). Responses of vegetation to a changing regime of disturbance: effects of feral pigs in a Californian coastal prairie. Ecogr.

[CR62] Kreith M (2007) Wild pigs in California: the issues. University of California Agricultural Issues Center, AIC Issues Brief. http://www.agmrc.org/media/cms/AgMRC_IB33v3_13C1D662ADDAE.pdf

[CR63] Loomis JB, Walsh RG (1997) Recreation economics decisions: comparing benefits and costs. Venture Publishing

[CR64] Loss SR, Will T, Marra PP (2013). The impact of free-ranging domestic cats on wildlife of the United States. Nat Commun.

[CR65] Love JD (2013) Robert Anthony Burrough, #1443625 versus Brad Livingston, et al. Civil Action (6)

[CR67] Lowe S, Browne M, Boudjelas S, DePoorter M (2000). 100 of the world’s worst invasive alien species: a selection from the global invasive species database.

[CR68] Mack RN, Simberloff D, Mark Lonsdale W, Evans H, Clout M, Bazzazz FA (2000). Biotic invasions: causes, epidemiology, global consequences, and control. Ecol Appl.

[CR69] Marbuah G, Gren IM, McKie B (2014). Economics of harmful invasive species: a review. Diversity.

[CR70] Margolis M, Shogren JF, Fischer C (2005). How trade politics affect invasive species control. Ecol Econ.

[CR71] Mayer JJ (2013) Wild pig attacks on humans. In: Armstrong JB, Gallaghher GR (eds) 15th Wildlife damage management conference. http://bit.ly/2dK07AQ

[CR72] Mayer J, Johns PE (2011) Characterization of wild pig vehicle collisions. Washington Savannah River Company (TABLE)

[CR73] McAusland C, Costello C (2004). Avoiding invasives: trade-related policies for controlling unintentional exotic species introductions. J Environ Econ Manag.

[CR74] McNeely JA (2001). Global strategy on invasive alien species.

[CR75] MEF U (2013) Beef, pork exports set new records in 2012. In U.S. Meat Export Federation

[CR76] MEF U (2014) Total U.S. pork exports. In U.S. Meat Export Federation

[CR77] Mengak MT (2012) Georgia wild pig survey: final report. Warnell School of Forestry and Natural Resources, University of Georgia

[CR78] Miller RE, Blair PD (2009) Foundations of input–output analysis. In: Input–Output analysis: foundations and extensions. Cambridge University Press, Cambridge, England, pp 10–68

[CR79] Miller RS, Farnsworth ML, Malmberg JL (2013). Diseases at the livestock–wildlife interface: status, challenges, and opportunities in the United States. Prev Vet Med.

[CR81] Miller R, Sweeney S, Slootmaker C, Grear D, DiSalvo P, Kiser D, Shwiff SA (2017) Cross-species transmission potential between wild pigs, livestock, poultry, wildlife, and humans; implications for disease risk management in North America. In press at Nature Scientific Reports 10.1038/s41598-017-07336-z PMC5552697 28798293

[CR82] Müller T, Hahn EC, Tottewitz F, Kramer M, Klupp BG, Mettenleiter TC, Freuling C (2011). Pseudorabies virus in wild swine: a global perspective. Adv Virol.

[CR83] National Feral Swine Damage Management Program (2019) Unpublished. (FIGURE)

[CR84] National Invasive Species Council (2001) Meeting the invasive species challenge: national invasive species management plan. 80

[CR85] No EO. 13112. (1999) National Invasive Species Council, Feb 3

[CR86] Ober HK, Edmondson GR, Giuliano WM (2011) Farmer perceptions of wildlife damage to row crops in North Florida. Department of Wildlife Ecology and Conservation, Florida Cooperative Extension Service, Institute of Food and Agricultural Sciences, University of Florida. http://edis.ifas.ufl.edu/uw356

[CR88] Pedersen K, Pabilonia KL, Anderson TD, Bevins SN, Hicks CR, Kloft JM, Deliberto TJ (2015). Widespread detection of antibodies to Leptospira in feral swine in the United States. Epidemiol Infect.

[CR89] Pederson K, Bauer NE, Olsen S, Arenas-Gamboa AM, Henry AC, Sibleys TD, Gidlewski T (2017a) Identification of *Brucella* spp. In feral swine (*Sus scrofa*) at abattoirs in Texas, USA. Zoonosis Public Health, pp 1–8 10.1111/zph.12359 28391650

[CR90] Pederson K, Bauer NE, Rodgers S, Bazan LR, Mesenbrink BT, Gidlewski T (2017). Antibodies to various zoonotic pathogens detected in feral swine (*Sus scrofa*) at Abattoirs in Texas, USA. J Food Prot.

[CR91] Perrings C, Williamson M, Barbier EB (2002). Biological invasion risks and the public good: an economic perspective. Conserv Ecol.

[CR92] Pimentel D (2007) Environmental and economic costs of vertebrate species invasions into the United States. Manag Vertebr Invasive Species 38. http://digitalcommons.unl.edu/nwrcinvasive/38

[CR93] Pimentel D, Lach L, Zuniga R, Morrison D (2000). Environmental and economic costs of nonindigenous species in the United States. Bioscience.

[CR94] Pimentel D, Zuniga R, Morrison D (2005). Update on the environmental and economic costs associated with alien-invasive species in the United States. Ecol Econ.

[CR95] Pineda-Krch M, O’Brien JM, Thunes C, Carpenter TE (2010). Potential impact of introduction of foot-and-mouth disease from wild pigs into commercial livestock premises in California. Am J Vet Res.

[CR96] Robeson MS, Khanipov K, Golovko G (2018). Assessing the utility of metabarcoding for diet analyses of the omnivorous wild pig (*Sus scrofa*). Ecol Evol.

[CR97] Rollins D (1998). Statewide attitude survey on feral hogs in Texas.

[CR98] Saito M, Koike F, Momose H, Mihira T, Uematsu S, Ohtani T, Sekiyama K (2012). Forecasting the range expansion of a recolonising wild boar Sus scrofa population. Wildl Biol.

[CR99] Sala OE, Chapin FS, Armesto JJ (2000). Global biodiversity scenarios for the year 2100. Science.

[CR100] Saunders G, Bryant H (1988). The evaluation of a feral pig eradication program during a simulated exotic disease outbreak. Aust Wildl Res.

[CR101] Schlaepfer MA, Sax DF, Olden JD (2011). The potential conservation value of non-native species. Conserv Biol.

[CR102] Seward NW, VerCauteren KC, Witmer GW, Engeman RM (2004) Feral swine impacts on agriculture and the environment. Sheep Goat Res J 12

[CR103] Shwiff SA, Gebhardt K, Kirkpatrick KN, Shwiff SS (2010). Potential economic damage from introduction of Brown Tree Snakes, Boiga irregularis (Reptilia: Colubridae), to the Islands of Hawai’i. Pac Sci.

[CR104] Shwiff SA, Carlson JC, Glass JH (2012). Producer survey of bird-livestock interactions in commercial dairies. J Dairy Sci.

[CR105] Shwiff SA, Kirkpatrick KN, DeVault TL, Shwiff SS (2015). Bioeconomic modeling of double crested cormorant impacts to a recreational fishery. Hum–wildl Interact.

[CR106] Shwiff SA, Ernest KL, Degroot SL, Anderson AM, Shwiff SS (2017a) The economic impact of blackbird damage to crops. In: Ecology and management of blackbirds (Icteridae) in North America. CRC Press, pp 207–216

[CR107] Shwiff S, Shwiff S, Holderieath J, Haden-Chomphosy W, Anderson A (2017b) Economics of invasive species damage and damage management. In: Ecology and management of terrestrial vertebrate invasive species in the United States. CRC Press, pp. 35–60

[CR108] Siemann E, Carrillo JA, Gabler CA, Zipp R, Rogers WE (2009). Experimental test of the impacts of feral hogs on forest dynamics and processes in the southeastern US. For Ecol Manage.

[CR109] Singleton G (2003). Impacts of rodents on rice production in Asia.

[CR110] Snow RW, Krysko KL, Enge KM, Oberhofer L, Walker-Bradley A, Wilkins L (2007) Introduced populations of Boa constrictor (Boidae) and Python molurus bivittatus (Pythonidae) in southern Florida. In: Henderson RW, Powell R (eds) Biology of boas and pythons. Utah, USA pp 365–386

[CR111] Spencer PB, Hampton JO (2005). Illegal translocation and genetic structure of feral pigs in Western Australia. J Wildl Manag.

[CR112] Sweitzer RA, McCann BE (2007) Natural areas ecological damage and economic costs survey report (survey report). University of North Dakota, pp 1–36

[CR113] Tolleson DR, Pinchak WE, Rollins D, Hunt LJ (1995) Feral hogs in the rolling plains of Texas: perspectives, problems, and potential. In: Great plains wildlife damage control workshop proceedings, p 454 (TABLE)

[CR114] Treyz GI, Rickman DS, Shao G (1991). The REMI economic-demographic forecasting and simulation model. Int Reg Sci Rev.

[CR115] Trout D, Gomez TM, Bernard BP, Mueller CA, Smith CG, Hunter L, Kiefer M (1995). Outbreak of brucellosis at a United States pork packing plant. J Occup Eviron Med.

[CR116] U.S. Fish & Wildlife Service (2012a) Chesapeake Bay nutria eradication project. http://www.fws.gov/chesapeakenutriaproject/FAQs.html

[CR117] U.S. Fish & Wildlife Service (2012b) The cost of invasive species. January 2012 factsheet. http://www.fws.gov/home/feature/2012/pdfs/CostofInvasivesFactSheet.pdf

[CR118] U.S. Fish & Wildlife Service (2012c) The economic cost of large constrictor snakes. January 2012 factsheet. http://www.fws.gov/home/feature/2012/pdfs/EconImpact.pdf

[CR119] USDA-APHIS-WS (2010) Nutria an invasive rodent. October 2010 Factsheet. https://www.aphis.usda.gov/publications/wildlife_damage/content/printable_versions/fs_nutria10.pdf

[CR120] USDA-APHIS-WS (2013) Feral swine: damage and disease threats

[CR121] USDA-ERS (2013) Home/topics/animal products/cattle & beef/statistics & information

[CR123] USGS (2013) Economic damages from the brown tree snake on Guam. http://www.fort.usgs.gov/resources/education/bts/impacts/economic.asp. Accessed 7 Aug 2013

[CR124] Vilà M, Basnou C, Pyšek P (2010). How well do we understand the impacts of alien species on ecosystem services? A Pan-European, cross-taxa assessment. Front Ecol Environ.

[CR125] Ward MP, Laffan SW, Highfield LD (2007). The potential role of wild and feral animals as reservoirs of foot-and-mouth disease. Prev Vet Med.

[CR126] Ward MP, Laffan SW, Highfield LD (2009). Modelling spread of foot-and-mouth disease in wild white-tailed deer and feral pig populations using a geographic-automata model and animal distributions. Prev Vet Med.

[CR127] Warner KD, Kinslow F (2013). Manipulating risk communication: value predispositions shape public understandings of invasive species science in Hawaii. Public Underst Sci.

[CR128] Weber WJ (1979). Health hazards from pigeons, starlings, and English sparrows: diseases and parasites associated with pigeons, starlings, and English sparrows which affect domestic animals.

[CR129] Weeks P, Packard J (2009). Feral hogs: Invasive species or nature’s bounty?. Hum Organ.

[CR130] Westenbroek T (2011) Letter to P. Anderson. Estimate of damage due to feral swine. Cornell University Cooperative Extension, Sullivan County

[CR131] Wilcove DS, Rothstein D, Dubow J, Phillips A, Losos E (1998). Quantifying threats to imperiled species in the United States. Bioscience.

[CR132] Williams CL, Johnston JJ (2004). Using genetic analyses to identify predators. Sheep Goat Res J.

[CR133] Witmer GW, Sanders RB, Taft AC (2003) Feral swine—Are they a disease threat to livestock in the United States? USDA National Wildlife Research Center—Staff Publications 292

[CR134] World Trade Organization (1994). Description of the agreement on the application of SPS measures.

[CR135] Yang X, Huang W, Tian B, Ding J (2014). Differences in growth and herbivory damage of native and invasive kudzu (*Peuraria montana* var. lobata) populations grown in the native range. Plant Ecol.

[CR136] Zhao Z, Wahl TI, Marsh TL (2006). Invasive species management: foot-and-mouth disease in the US beef industry. Agric Res Econ Rev.

